# Behavioral and Neural Correlates of Cognitive Training and Transfer Effects in Stroke Patients

**DOI:** 10.3389/fneur.2020.01048

**Published:** 2020-09-15

**Authors:** Eliane C. Miotto, Paulo R. Bazán, Alana X. Batista, Adriana B. Conforto, Eberval G. Figueiredo, Maria da Graça M. Martin, Isabella B. Avolio, Edson Amaro, Manoel J. Teixeira

**Affiliations:** ^1^Department of Neurology, University of São Paulo, São Paulo, Brazil; ^2^Institute of Radiology, LIM-44, University of São Paulo, São Paulo, Brazil

**Keywords:** stroke, cognitive training, episodic memory, semantic organization strategies, fMRI

## Abstract

Stroke lesions are frequently followed by cognitive impairments. Cognitive training is a non-pharmacological intervention that can promote neural compensation mechanisms and strategies to remediate cognitive impairments. The aims of this study were: (1) To investigate the cognitive performance, generalization effects, and neural correlates of semantic organization strategy training (SOST) in patients with chronic left frontoparietal stroke and healthy controls (HC); and (2) to compare the behavioral effects and neural correlates of SOST with an active control psychoeducation intervention (PI). In this randomized controlled study, all participants were randomly allocated into two groups, one group received SOST, and the other received PI intervention. Participants underwent two fMRI sessions, one prior and the other, after intervention. In each fMRI session, images were obtained during memory encoding task using a list of semantically related words. We found improved post-intervention memory performance in participants that received SOST (both patients and controls), indicated by number of words recalled, word clustering scores, and performance in a generalization task. The fMRI analysis revealed negative correlation between task performance and regions of the default-mode network. These results suggest that cognitive training using semantic organization strategy can improve episodic memory performance and promote potential functional neuroplasticity in patients with ischemic stroke lesions.

**Clinical Trial Registration:** www.ClinicalTrials.gov, identifier: NCT03644290.

## Introduction

Cognitive impairment occurs in more than one third of patients with stroke and persists in many individuals for years, producing long-term disabilities (1, 2). Up to half of patients with cognitive deficits following stroke show significant impairment in episodic memory (3, 4). Episodic memory is a system implicated in the capacity to learn and recall past information or events (5). Cognitive rehabilitation is a traditional non-pharmacological treatment approach directed at the restoration of cognitive activity or the acquisition of efficient strategies to compensate for impaired cognitive function, particularly episodic memory (6, 7). In patients with memory impairments due to vascular and traumatic brain lesions, cognitive interventions have been recommended as a practice standard, including the use of internalized strategies (e.g., verbal association, visual imagery, etc.) and external memory compensation (e.g., cellphones, notebooks, diaries) (6–8). Recent studies demonstrated that patients with vascular lesions can benefit from different cognitive interventions including face-name training to remember people's names and repetition-lag memory training, developed to increase recollection as opposed to familiarity in recognition memory tasks (9, 10). There is also recent evidence of memory and attention improvement after computerized cognitive training and telehealth options for remote delivery of compensatory memory skills training after stroke (11, 12).

Semantic organization strategy training (SOST) is a cognitive intervention designed to recruit executive functions, semantic categorization, working memory, engaging regions of frontoparietal network, particularly in the left hemisphere due to verbal stimuli processing (13–15). This cognitive training (CT) intervention is based on the application of semantic organization strategy to word-lists in order to improve free verbal episodic memory recall and to enhance encoding by grouping words together that belong to the same category. Previous studies using SOST showed improvement in episodic memory in healthy adult individuals, patients with left frontal glioma excisions and mild cognitive impairment (14–16). Nevertheless, the underlying brain mechanisms related to cognitive interventions in patients with stroke remain largely unknown. In particular, no study has investigated, as yet, the effects of CT using SOST in patients with stroke in the left frontoparietal hemisphere.

Neuroimaging methods, particularly functional magnetic resonance imaging (fMRI), have been used to investigate the neural substrates underlying cerebral plasticity after cognitive training in a limited number of studies in patients with traumatic brain injury (17–20). In patients with vascular lesions or stroke, one study demonstrated changes in activation after training in default-mode network regions, such as the posterior cingulate cortex, precuneus, and angular gyrus, as well as in lateral occipital and temporal regions in parallel to behavior improvements (9).

Another study found memory and executive function improvement and increased resting-state functional connectivity of the hippocampus with the frontal lobe (right inferior, right middle, left middle, left inferior, and left superior frontal gyrus) and the left parietal lobe in a small sample of patients with heterogeneous stroke lesions after computerized cognitive training using the RehaCom software package (21). The authors associated these findings with mechanisms of brain compensation and cognitive recovery in patients who received cognitive training.

The investigation of the impact of individual CT interventions, such as SOST, visual imagery, etc., outside the context of multi-domain cognitive and holistic rehabilitation programs is highly relevant to understand the effectiveness of each specific approach and its brain mechanisms to plan cognitive rehabilitation programs in a more effective way. Yet, no study has explored the behavioral effects and neural correlates of SOST intervention in patients with stroke, particularly, involving the left frontoparietal brain regions known to affect episodic memory encoding due to reduced strategy and efficient executive processes application (13–15). As described above, SOST intervention is thought to recruit left frontoparietal network regions due to its verbal stimuli word-list presentation and semantic strategy application in order to improve encoding and verbal episodic memory recall. Previous studies using SOST were carried in patients with left frontal tumors and MCI people (15, 16). Nevertheless, no investigation has, so far, been conducted in stroke patients, especially with lesions in those areas of the left frontoparietal network thought to be involved in this strategy. To pursue this investigation, a sample of healthy control participants would be necessary in order to investigate specific cerebral metabolic changes and extent of improvement in behavior in patients with left frontoparietal stroke lesions in comparison to what is seen healthy subjects.

Therefore, the aims of the current study were: (1) to investigate the cognitive performance changes, generalization effects and neural correlates of SOST intervention in patients with left frontoparietal stroke and healthy controls; and (2) to compare the behavioral and transfer or generalization effects and neural correlates of SOST with an active control psychoeducation intervention (PI). We hypothesized that all participants, particularly the stroke patients (SP), would benefit from SOST in comparison to the control intervention, and that different neuronal brain mechanisms would be involved in patients with ischemic stroke lesions in relation to healthy controls.

## Materials and Methods

### Participants

**Figure 1:**
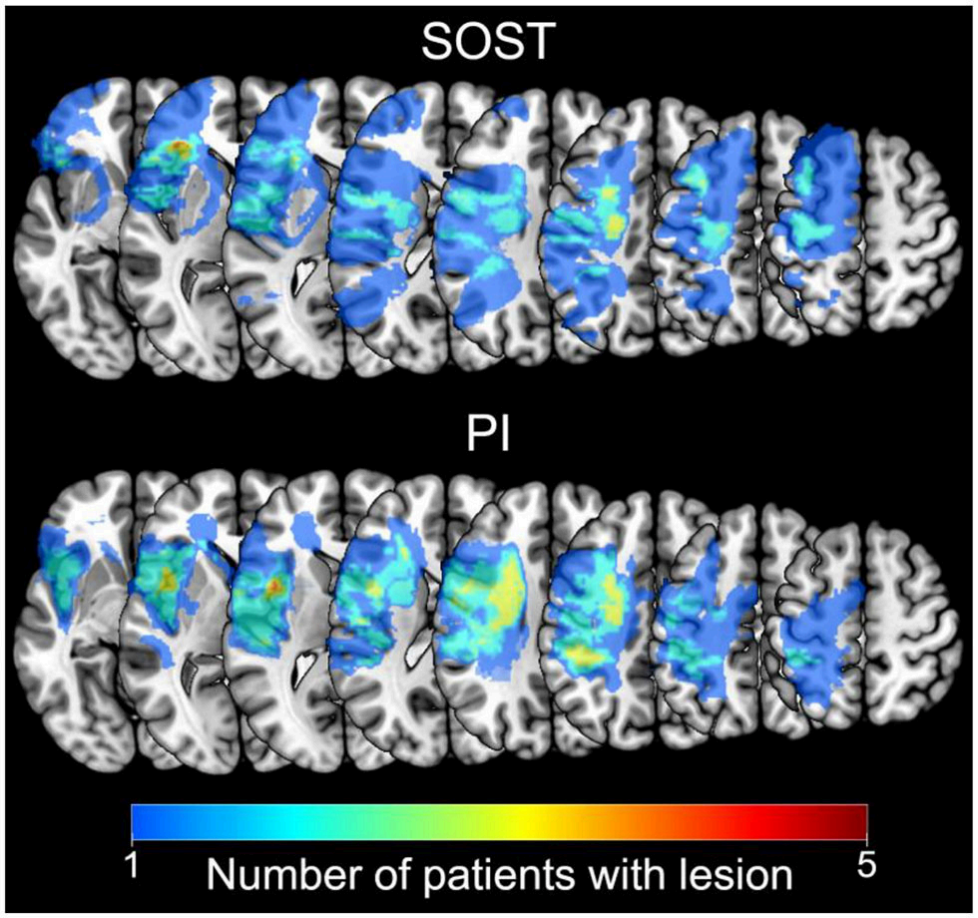
Lesion Maps. Superposition of patients' lesions represented by the number of subjects that had lesions in each voxel. Left hemisphere lesion maps are presented separately for each group: patients that received Semantic Organization Strategy Training (SOST; *n* = 7; top); and patients that received Psychoeducation Intervention (PI; *n* = 6; bottom). Images in neurological orientation.

**Figure 2:**
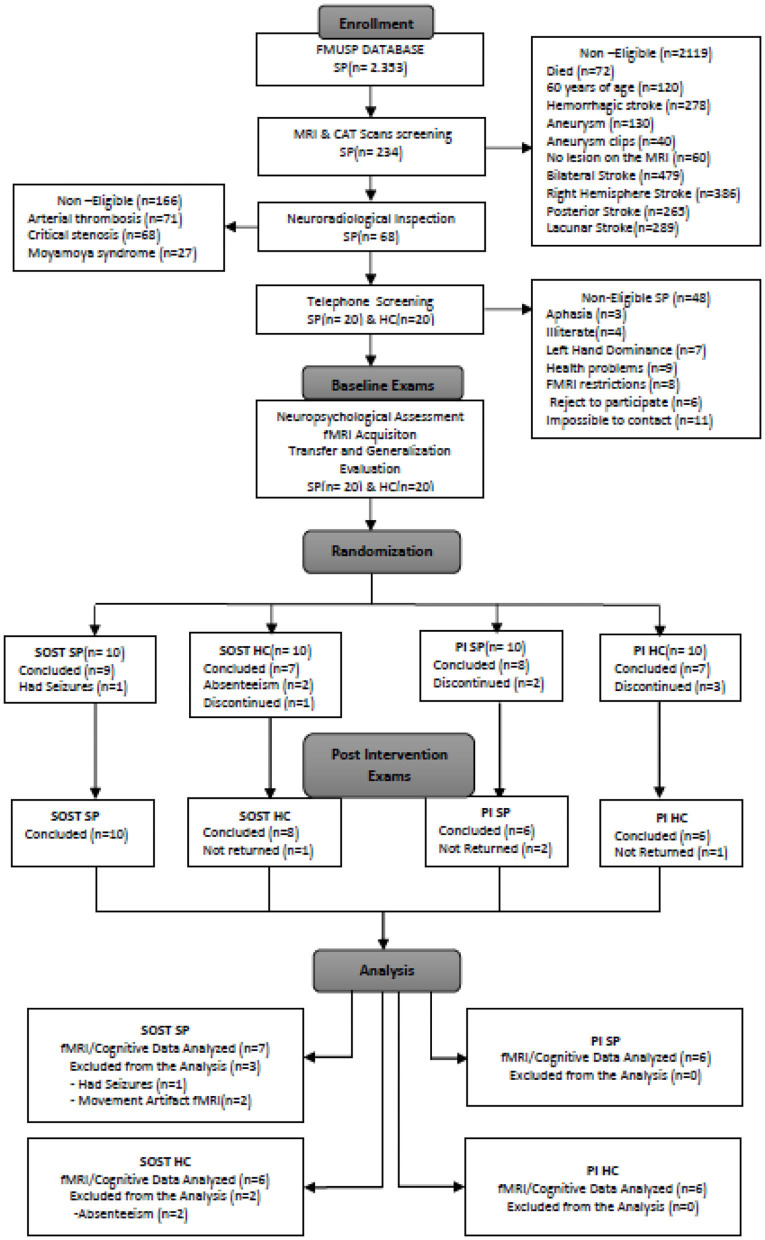
CONSORT flowchart of the recruitment and selection of the study participants. SP, Stroke patients; HC, Healthy Controls; fMRI, Functional Magnetic Resonance Imaging; SOST, Semantic Organization Strategy Training; PI, Psychoeducational Intervention.

A total of 25 participants, 13 SP and 12 healthy controls (HC), all right handed were included in the final analysis of this study. The SP were recruited from the Vascular Neurology Clinic at the Department of Neurology, Hospital das Clínicas, São Paulo University. We included adult patients with lesions only in the left hemisphere (Figure 1), who had suffered ischemic stroke more than 6 months before the fMRI session (average 4 years, range 1–13 years), were non-aphasic and free from other neurological or psychiatric disorder, as tested by neurological and neuropsychological evaluations. Exclusion criteria involved those with left temporo-occipital, hippocampal, parahippocampal, right hemisphere or bilateral lesions, critical stenosis, arterial thrombosis, hemorrhagic stroke, aneurysm, left-handedness, and more than 60 years of age to avoid confounding existing pathology, such as possible MCI due to Alzheimer's Disease (DA) and other neurodegenerative disorders. The subjects selection procedure is shown in the flowchart (Figure 2) adapted from the CONSORT diagram (22). This final sample was selected from a total of 2.353 stroke patient protocols screened from 2009 to 2017; 2.119 did not meet the study criteria based on their clinical records. The brain MR images of the remaining 234 SP were examined by neuroradiologists and only 68 patients met the study criteria for the telephone interview. After the telephone contact, 20 SP were enrolled in the study but only the cognitive and fMRI data of 13 SP were analyzed. Amongst the seven SP that were excluded, four did not finish the study, one had epileptic seizures during the intervention and two were excluded due to the movement artifacts on the post fMRI exam. Twenty HC were recruited from the community through social media advertisement to match the age and schooling profile of the SP.

However, six HC discontinued their participation and two HC were excluded due to their absence during the intervention and post-training evaluation. The main reason for this dropout in the HC group were difficulties in adjusting their work schedule in order to participate in the evaluation and intervention sessions since these sessions were time consuming and were performed during the business hours. Also, HCs showed reduced interest to continue the study since they had no cognitive impairment or complaints in everyday life. Thirteen right-handed SP (6 males) and 12 right-handed HC (4 males) were included in the current study. For patient screening, lesions were evaluated by blinded radiologists using clinically acquired fluid attenuated inversion recovery (FLAIR) and high-resolution T1 sequences. All subjects had either normal or corrected to normal standard vision, by using MRI-compatible eyeglasses. The HC participants were assessed by the same instruments as the SP and their brain MR images by the same neuroradiologists. This study was approved by the local ethics committee (CAPPesq 8839) and ClinicalTrials.gov Identifier: NCT03644290. All participants provided written informed consent, and this study was conducted in compliance with the Declaration of Helsinki.

### Procedure

All participants in the study underwent cognitive assessment by neuropsychologists to estimate their baseline cognitive functioning using the total estimated IQ (WAIS-III) calculated from the Vocabulary and Matrix Reasoning (23). Following the cognitive assessment, all participants carried out the pre-training fMRI exam using an episodic memory paradigm (see detailed description below) conducted by two different neuropsychologists and the biomedical team from the Department of Radiology. After imaging acquisition and in a separate day, all participants completed a baseline transfer and generalization ecological task and a brief metamemory questionnaire (BMQ), conducted by different neuropsychologists, which lasted 90 min. Subsequently and after the cognitive training sessions, all participants carried out the post-training fMRI exam using a similar and parallel episodic memory paradigm, generalization task and the BMQ, although with different stimuli.

The primary outcome measures included the post vs. pre-intervention differences in words recalled and semantic clustering scores related to the fMRI task developed to assess encoding during verbal episodic memory learning and semantic strategy application, which were the main purpose of the cognitive training (see more detailed description below and in previous studies 14–16 using the same paradigm). The semantic clustering scores were described as the number of consecutive recalls of two words from the same category. Secondary outcome measures were the transfer and generalization task scores (recall and clustering), memory capacity (based on the complaints questionnaire), and strategy application obtained from the BMQ to investigate the transfer of the strategies learned during cognitive training to a novel and ecological scenario and frequency of cognitive complaints and use of strategy (see below further description).

For the randomized controlled trial (RCT) procedure, volunteers were randomly assigned to SOST or PI by a random number generator from Microsoft Excel (RAND function—Microsoft Corporation). Each intervention consisted of three individual sessions of 90 min carried out within 2 weeks, with intervals of 2–3 days between sessions (see detailed description below). After the intervention, they completed an alternative version of the transfer and generalization ecological task to assess possible transfer and generalization effects of the intervention (see detailed description below). The post-intervention transfer and generalization ecological task was carried out within 3 days after the end of the intervention in a separate 90 min session. Therefore, all participants received five sessions in total, including two sessions of transfer and generalization pre and post-intervention and three sessions of SOST or PI. One week after the post-intervention generalization session, all participants completed the post-intervention fMRI acquisition.

The current study adopted a double-blind randomized controlled trial (RCT) approach (ClinicalTrials.gov Identifier: NCT03644290), in a way that participants and neuropsychologists who performed cognitive evaluations and fMRI data acquisition were blinded to the intervention type. In addition, the neuropsychologists for the fMRI acquisition were different and blinded to the results of the cognitive examination. Also, the interventions were conducted by three different neuropsychologists who were unaware of participant's diagnosis and results of the cognitive tests and outcome measures.

### Experimental Study Design

**Figure 3:**
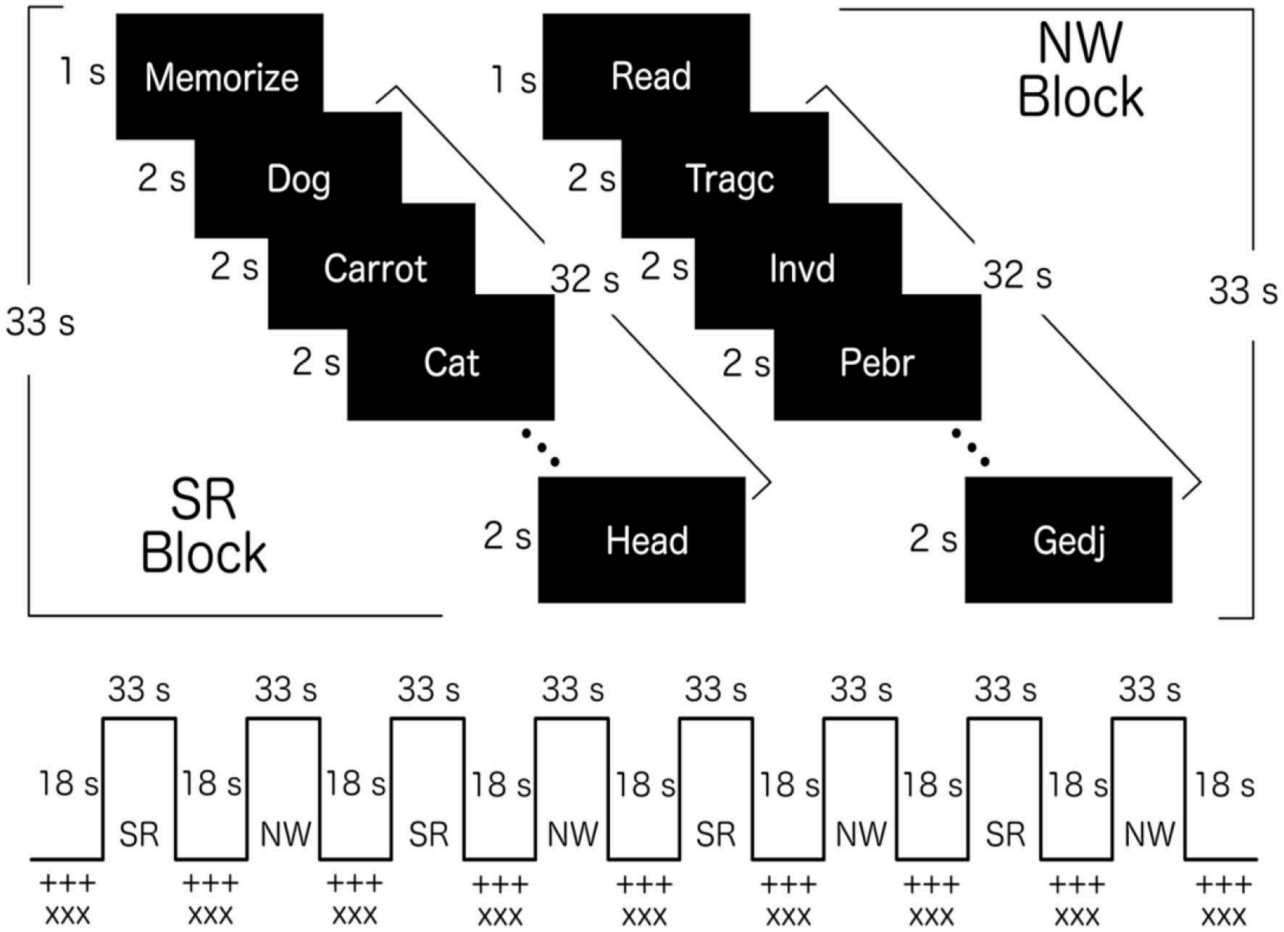
Word-List Learning Paradigm (WLLP). Schematic representation of WLLP showing the structure of each task block (top): Semantically related (SR) block; and non-words (NW) block. The words presented in the image are only illustrations. The experiments were performed with words in Portuguese. The fMRI block sequence is also presented (bottom) showing an example with SR block (participants were randomly assigned to start with SR block or with NW block).

All volunteers performed two fMRI sessions, one before, and one after intervention. In each session, participants completed a 426s “Word-List Learning Paradigm” (WLLP) sequence. During the WLLP (Figure 3), volunteers were presented with sequences of words to memorize, each shown for 2s, in blocks of 16 words, followed by a 18s baseline interval in which the symbols “+++++” or “XXXX” were presented in the center of the screen alternating every 2s. There were two types of task blocks: one with lists of semantically related (SR) words randomly presented and validated in previous studies (12–15) and another block with non-words (NW). The words in SR block were related in terms of four categories. The categories were different in the pre-training fMRI (general tools, gardening and lawn care items, flowers and footwear) and in the post-training fMRI (candy and sweets; sports; fighting styles and months of the year). No two words from the same category occurred consecutively within the list. As an example of the WLLP measure in the SR block, the 16 words included were: Eye, Penguin, Dustpan, Oyster, Detergent, Squid, Beaver, Finger, Cod, Sponge, Deer, Ankle, Bucket, Salmon, Nose, and Wolf. In this particular word-list we had four categories, namely Body Parts, Land Animals, Cleaning Supplies, and Water Animals. A more detailed description of the word-lists can be found in our previous studies (12–15).

Each task block started with an instruction presented for 1s: “Read” and “Memorize.” This unique paradigm allows for the use of semantic organization strategy in order to encode and recall the words in a more efficient way. The non-words were created from words used in the SR condition by replacing the first or final syllable, making the new word non-meaningful, but nevertheless readable (phonologically amenable to pronunciation). Both lists of stimuli contained the same number of letters and syllables. This procedure was based on a previous study (24).

Each task block was presented 4 times, and the baseline block was presented 9 times (before and after each task block). The task blocks were presented in an alternated order, and volunteers were randomly assigned to either start with SR block or with NW block. Right after the task run, participants were instructed to recall the words they memorized during the fMRI task (WLLP recall). Two scores were computed for each recall condition: the total number of words correctly recalled and a semantic clustering score. The semantic clustering scores were defined as the number of consecutive recalls of two words from the same category. For the primary and secondary outcome measures, the post vs. pre-intervention differences in words recalled and semantic clustering scores were calculated.

### Image Acquisition

FMRI images were obtained with a 3.0 Tesla Philips Achieva system with a receiver 32 channel head coil. Two structural sequences, a T1 weighted three-dimensional image (Time of repetition = 7 ms; Echo Time = 3.2 ms; Inversion Time = 900 ms; 180 sagittal slice; Field of view = 240 × 240 mm^2^, voxel size = 1 × 1 × 1 mm^3^) and a T2 weighted FLAIR image (Time of repetition = 11,000 ms; Echo Time = 130 ms; Inversion Time = 2,800 ms; 28 axial slices with thickness of 4.5 mm and gap of 0.5 mm; Field of view = 230 × 183.28,125 mm^2^ with a 356 × 210 matrix), were used for evaluation of the lesions and for registration into standard template. Also, a T2^*^-weighted echo planar sequence was used in the WLLP task sequence (Time of repetition = 3,000 ms; Echo Time = 30 ms; 40 axial slices with thickness of 3 mm; Field of view = 240 × 240 mm^2^ with an 80 × 78 matrix; 142 volumes).

### Transfer and Generalization Evaluation—Supermarket Generalization Task (SGT) and Brief Metamemory Questionnaire (BMQ)

In order to measure the effect of the semantic categorization strategy training on participant's everyday functioning scenario, an ecological generalization task was designed. The SGT consisted of a list of 12 supermarket items organized into 3 categories displayed on iPad (Apple Inc.). Each supermarket item was displayed with its respective name and colored picture. All participants were instructed to perform the ecological task considering a scenario in which they had to go to a supermarket to buy some items, such as chicken, lamb, cod and beef for “meat types” category; lettuce, carrots, spinach, and cucumber for “vegetables” category. All items that participants should memorize to buy at the supermarket were presented one at a time for 2 s and read out loud by them. Right after the presentation, the participants were asked to recall which items they remembered. Two alternative versions of the SGT were prepared and randomly assigned to each participant pre and post-intervention to minimize the effect of task and order.

All participants answered the BMQ after recall of the SGT. The BMQ has eight questions about daily memory problems and mnemonic strategies. For the four memory problem questions the participants had to estimate the magnitude of their memory difficulties related to (1) recall the name of persons they met (2) remember items from a shopping list (3) recall certain words during conversations and (4) remember the information they read in newspapers, books or texts. The ratings were converted into numerical values using a five-point scale: (1) no difficulty, (2) mild difficulty, (3) moderate difficulty, (4) serious difficulty, and (5) very serious difficulty. The total sum of the four memory problems ratings were used as a verbal memory difficulty index. This was then converted to a capacity index, calculated as the maximum difficulty score sum minus the memory difficulty index from that session (participants with 0 difficulty would have the maximum score in the capacity index, and participants with the maximum difficulty would have a 0 score in the capacity index). Moreover, the participants were asked to rate in a scale of 0 (no strategy use) to 10 (highly frequent strategy use) their knowledge and use of memory strategy, including the semantic organization strategy, for each of the four memory problems listed above. The sum of these four strategy ratings were used as a verbal memory strategy index.

### Intervention Training Protocol

All participants were randomly assigned to SOST or PI individual interventions by a random number generator from Microsoft Excel (RAND function—Microsoft Corporation), as previously described, and the sessions were carried out by three different neuropsychologists blinded to the participant outcome measures results. For the SOST, subjects were instructed and trained to organize lists of words into categories and to retrieve them according to their category. In each session, words and their corresponding pictures were presented using iPad and trained in three stages following a hierarchical difficulty level. Participants were asked to, firstly, identify the groups to which the words belonged to and then, to recall the words grouped into each category (e.g., furniture, means of transport, garments, etc.). In all 3 hierarchical stages, the words were repeated and subsequently recalled by the participants until they reached 80% of correct responses. The difficulty level of the training was increased by adding new words from new categories to the task, until the last level of training difficulty was reached. The training started with 6 words and their corresponding pictures randomly grouped into two categories. Subsequently, 6 new words and pictures were added to the previous list: one from each of the previous categories, and four from a new one. At this level of training, subjects had to remember 12 words from 3 categories. In the third and last level, a set of 4 new words and their corresponding pictures from a new category were added, and the subject had to recall 16 words grouped into four different categories.

For the PI controlled intervention, participants received comprehensive stroke information sessions using iPad including text and images on risk factors, diet, lifestyle, types of stroke, cognitive and motor symptoms, treatment and recovery. At the end of each session, they had to answer questions related to the information they learned. Cognitive strategies were not provided during the PI sessions. The sessions were carried out using the same format and duration as the SOST intervention and by the same neuropsychologists who conducted the SOST.

### Data Analysis

#### Behavioral Data Analysis

Based on the WLLP, free recall results were obtained after each fMRI session and two metrics were generated: the total correct number of words recalled and a semantic clustering score. As described above, the semantic clustering score was defined as the number of times two consecutive words were recalled from the same category. There was also a free recall measure for the SGT based on the total correct number of items recalled and a semantic clustering score. For the statistical procedures we used JASP 0.12 (25) and R 3.6.2 (26). In order to evaluate group paring, normality was assessed by Shapiro-Wilk test and non-normal data were log transformed and one-way ANOVAs were used to analyze group differences regarding age, schooling and IQ performance. Also, a *t-*test was used to compare the lesion volume between SP. To evaluate intervention, the post vs. pre-intervention differences were calculated for the WLLP total recall and clustering scores, for the SGT total recall and clustering scores, and for the BMQ strategy and capacity scores. Two type III MANOVAs (using “car” library in R) evaluating group (SP vs. HC), intervention (SOST vs. PI) and the interaction between group and intervention were performed: one using the scores from fMRI task (WLLP recall and clustering scores), and the other with scores not-related to fMRI task (SGT and BMQ scores). Given our sample size, there were not enough degrees of freedom to use all variables in a single MANOVA. Also, there were missing data from SGT and BMQ, but the fMRI dataset was complete, further suggesting splitting these scores. As *post-hoc* tests for significant results in the MANOVAs, ANOVAs with each of the six scores were calculated, and the *p-values* from these *post-hoc* tests were FDR corrected. Also, to further evaluate if the use of strategies were associated with better capacity (lower complaints), a correlation test was calculated between the post-pre intervention differences in strategy and in capacity. Results were considered significant when reaching a threshold of *p* < 0.05 (after FDR-adjusted *p-value* correction in the case of *post-hoc* tests).

#### fMRI Preprocessing

Data were preprocessed following standard guidelines implemented in FSL 6.0.0 package (www.fmrib.ox.ac.uk/fsl) (27): image realignment (using MCFLIRT); slice timing correction; spatial smoothing with a 5 mm full width at half maximum Gaussian kernel; high-pass filtering, with a cutoff of 120s period for task runs. After image realignment, the images were subjected to a motion and artifact evaluation using the ART toolbox (http://www.nitrc.org/projects/artifact_detect/). Volumes with motion above 0.9 mm or with high signal change (above 5 z-transformed), were marked for scrubbing. Volunteers that had more than a quarter of the volumes marked for scrubbing were excluded from the study. To allow group level analyses, images were registered to the MNI-152 2 mm template in two steps: first, T1 structural images were registered to the template, with a non-linear registration procedure (using FNIRT); second, the functional images were registered to the T1 images, with a linear registration (using epi_reg command in FLIRT), and then passed to standard template by applying the registration generated in the first step. To avoid distortions due to brain lesions, lesion masks were generated and used to remove their weight in the estimation of the non-linear T1 to template registration (28).

#### fMRI Task-Based Analysis

To evaluate the brain regions associated with the task in the first-level analysis (individual level), we used General Linear Model (GLM) with the expected hemodynamic response during each type of block. The two blocks were modeled separately using a boxcar function convolved with a double-gamma canonical hemodynamic response function. To avoid motion-induced artifacts, the motion parameters calculated during realignment procedure were used as regressors in the GLM analysis. Further, additional scrubbing variables were included in the GLM, based on the volumes marked by ART toolbox with high motion (above 0.9 mm) or abrupt fMRI signal change (above 5 z-transformed). Prior to GLM analysis, pre-whitening was performed using semi-parametric estimation of residuals autocorrelation, as implemented in FILM routines (29). In this first-level analysis, the contrast of SR blocks against baseline blocks was estimated and then used in higher-level analyses. This contrast offers higher statistical power compared to SR against NW blocks, although with less specificity to the memory effects.

For higher-level analyses, separate ANOVAs were calculated for each group (SP and HC), modeling run effects as well as the interaction between run and intervention type (following FSL guidelines: https://fsl.fmrib.ox.ac.uk/fsl/fslwiki/GLM). Also, an ANOVA with all subjects, evaluating the group and intervention type effects in the difference between post and pre-training brain activity was performed. For this, the post-pre intervention differences for each subject were estimated as fixed effects in an intermediate step of analysis. Further, to better characterize the blood-oxygen-level-dependent (BOLD) response to SR task in each subgroup (PI HC, PI SP, SOST HC, and SOST SP), averages of brain activity during SR blocks were calculated for each run (pre and post intervention) separately. Prior to the thresholding step in all higher-level analyses, binary maps formed by the sum of the lesion maps from individuals included in each analysis were used to restrict group maps to preserved brain tissue. All statistical maps were generated with gaussian random field-based cluster inference, using voxel threshold of *Z* > 2.3 and cluster corrected threshold of *p* < 0.05, to provide a balance between statistical power and false-positive control.

## Results

### Behavioral Data

There were no significant differences between groups in terms of age [SOST (HC mean: 33.5, SD:17.0, and SP mean: 30.0,SD: 13.5), PI (HC mean:43.2, SD: 9.7, and SP PI mean: 42.3, SD: 8.6)], years of education [SOST (HC mean: 10.7, SD: 3.6, and SP mean: 12.3, SD: 1.9), PI (HC mean: 11.3, SD: 4.1, and SP mean: 11.8, SD: 1.3)] and IQ performance [SOST (HC mean: 99.0, SD: 16.2, and SP mean: 95.6, SD: 7.3), PI (HC mean:93.0, SD: 6.1, and SP mean:95.0, SD: 10.7)]. Also, lesion volumes did not differ amongst stroke patient groups (SOST mean: 29.0, SD: 20.1, and PI mean: 34.8, SD: 34.7).

**Figure 4:**
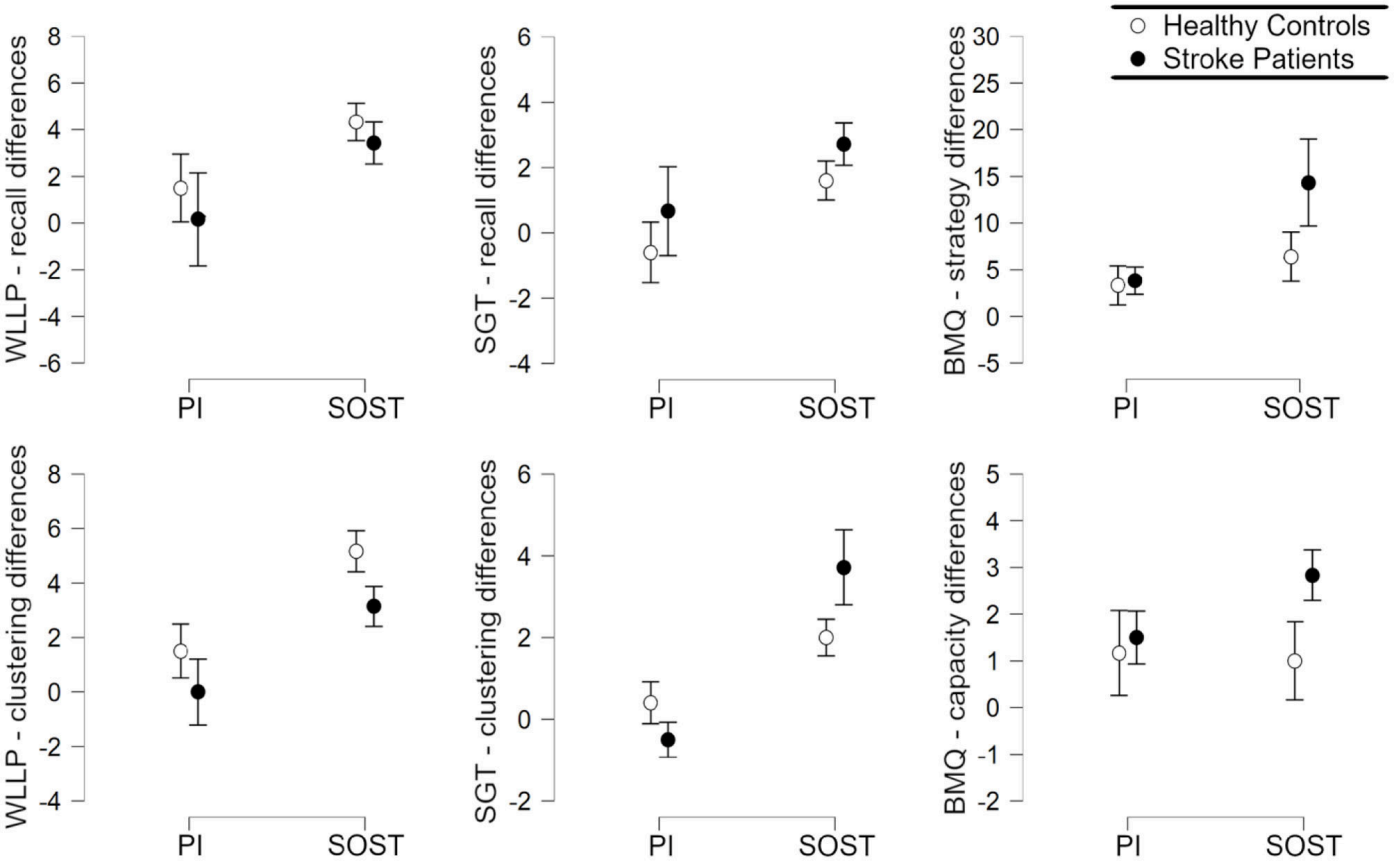
Plots of the Post vs. pre intervention average differences, with standard error bars, according to group (Healthy Controls; Stroke patients) and intervention (SOST, Semantic Organization Strategy Training; PI, Psychoeducation Intervention), for each of the 6 dependent variables evaluated: Word-List Learning Paradigm (WLLP) recall; WLLP clustering score; Supermarket Generalization Task (SGT) recall; SGT clustering score; brief metamemory questionnaire (BMQ) strategy use; and BMQ capacity.

Behavioral mean data and standard deviation for each group are presented separately in Table 1. Regarding WLLP performance (fMRI task), the MANOVA indicated significant intervention effect [*F*_(2,20)_ = 6.46, *p* = 0.0069], but no group [*F*_(2,20)_ = 1.94, *p* = 0.17], nor interaction effects [*F*_(2,20)_ = 0.16, *p* = 0.85]. The *post-hoc* ANOVAs indicated that the intervention effect was significant for both the total recall differences [*F*_(1,21)_ = 5.16, FDR-corrected *p* = 0.046] and the clustering score differences [*F*_(1,21)_ = 13.38, FDR-corrected *p* = 0.0044]. These results were produced by higher post vs. pre-intervention differences in SOST groups (Figure 4).

**Table 1:** Demographic and behavioral results for stroke patients (SP) and healthy controls (HC).

	**Semantic organization strategy training**						**Psychoeducational intervention**					
	**Pre-intervention**			**Post-intervention**			**Pre-intervention**			**Post-intervention**		
	**SP**	**HC**	**Total**	**SP**	**HC**	**Total**	**SP**	**HC**	**Total**	**SP**	**HC**	**Total**
WLLP total recall	6.6 (4.1)	7.8 (3.2)	7.2 (3.6)	10.0 (3.5)	12.2 (3.0)	11.0 (3.3)	7.7 (3.1)	6.5 (2.0)	7.1 (2.6)	7.8 (4.7)	8.0 (5.3)	7.9 (4.8)
WLLP semantic cluster	1.7 (1.6)	2.5 (2.8)	2.1 (2.2)	4.9 (2.7)	7.7 (2.9)	6.2 (3.1)	3.5 (2.3)	2.0 (1.7)	2.8 (3.1)	3.5 (3.7)	3.5 (3.0)	3.5 (3.2)
BMQ memory capacity index	9.2 (3.4)	10.8 (2.2)	9.9 (2.4)	12.0 (2.6)	11.8 (2.5)	11.9 (2.4)	8.0 (2.4)	8.8 (3.5)	8.4 (2.9)	9.5 (2.7)	10.0 (2.9)	9.8 (2.7)
BMQ memory strategy index	10.2 (7.3)	15.6 (10.2)	12.6 (8.7)	24.5 (5.8)	22.0 (6.1)	23.4 (5.8)	18.5 (4.0)	18.8 (10.7)	18.7 (7.7)	22.3 (4.6)	22.2 (8.2)	22.3 (6.3)
SGT total recall	7.3 (1.8)	8.0 (2.0)	7.6 (1.8)	10.0 (1.6)	9.6 (1.8)	9.8 (1.6)	7.8 (2.6)	7.6 (1.5)	7.7 (2.1)	8.5 (2.7)	7.0 (1.0)	7.8 (2.1)
SGT semantic cluster	1.1 (1.1)	2.4 (2.4)	1.7 (1.8)	4.9 (2.5)	4.4 (3.3)	4.7 (2.7)	2.2 (1.2)	1.4 (0.5)	1.8 (1.0)	1.7 (0.5)	1.8 (1.1)	1.7 (0.8)

*Mean results followed by standard deviation in parentheses are displayed for Word List Learning Paradigm (WLLP), Supermarket Generalization Task (SGT), and Brief Metamemory Questionnaire (BMQ)*.

Similarly, the MANOVA with the SGT and BMQ scores showed a main effect of intervention [F_(4, 15)_ = 5.50, *p* = 0.0063], but the main effect of group [*F*_(4, 15)_ = 1.09, *p* = 0.39] and the interaction effect between group and intervention [*F*_(4, 15)_ = 1.27, *p* = 0.32] were not significant. The *post-hoc* ANOVAs showed that the intervention effect was significant for STG recall difference [*F*_(1, 19)_ = 4.97, FDR-corrected *p* = 0.046], SGT clustering score difference [*F*_(1, 19)_ = 18.06, FDR-corrected *p* = 0.0026], and BMQ strategy use difference [*F*_(1, 19)_ = 5.06, FDR-corrected *p* = 0.046], but not for BMQ capacity difference [*F*_(1, 19)_ = 0.65, FDR-corrected *p* = 0.43]. These results were associated with higher differences in SOST groups (Figure 4). Also, there was a significant correlation between BMQ strategy use differences and BMQ capacity differences (*r* = 0.56, *p* = 0.0056).

### fMRI Task Results

#### Average BOLD Responses

**Figure 5:**
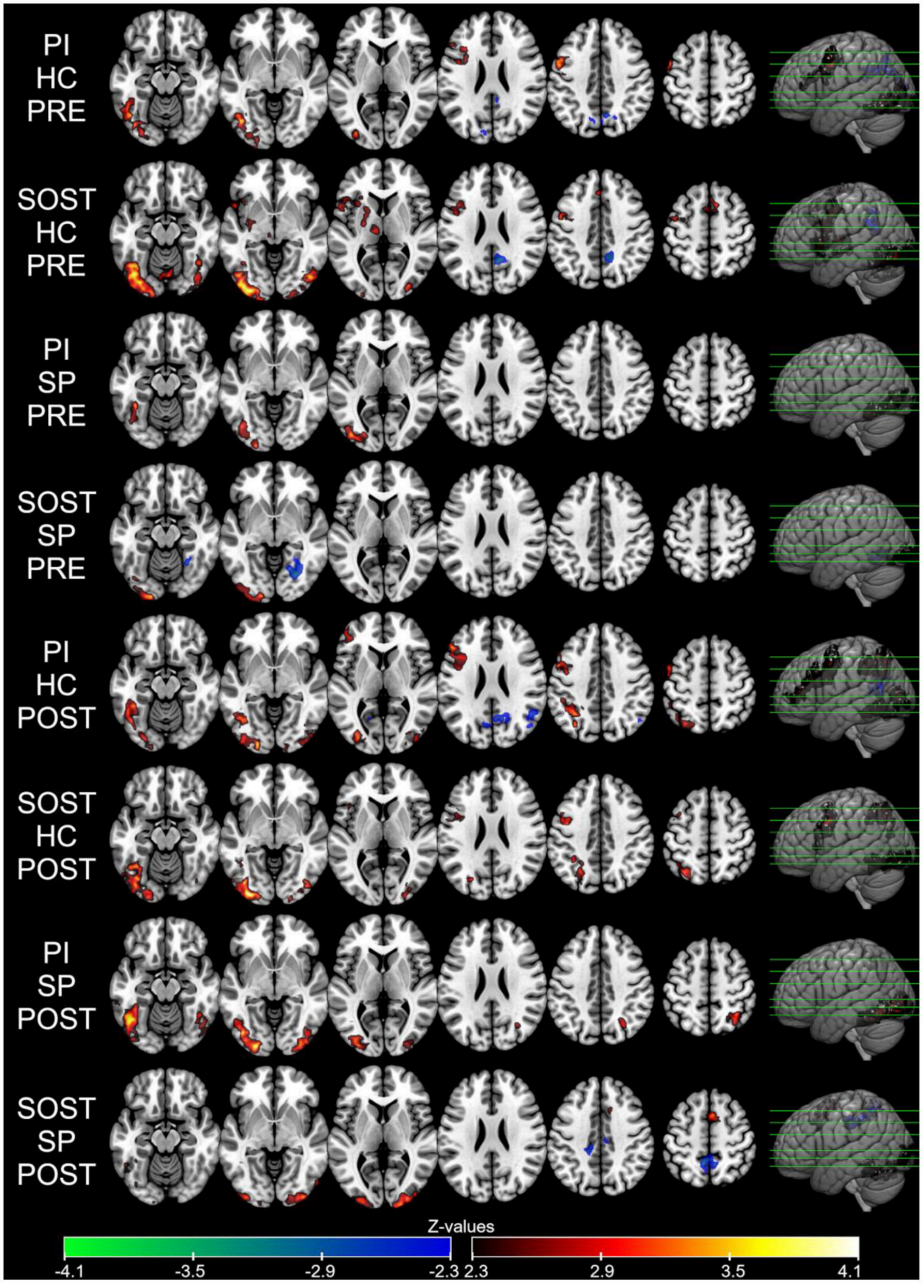
Average BOLD responses in SR task block for each group in each run (Pre and Post-intervention) presented as *Z-*values (neurological orientation). Voxel threshold of *Z* > 2.3 and cluster corrected threshold of *p* < 0.05. PI, Psychoeducation Intervention; SOST, Semantic Organization Strategy Training; HC, Healthy Controls; SP, Stroke Patients.

The average increase (activation) and decrease (deactivation) in BOLD signal associated with WLLP task are presented in Figure 5 and in Table 2. All groups in all runs presented activation in the primary visual cortex. In the pre intervention run, this was mainly observed in the left visual cortex, but after interventions, it was significant in both hemispheres in all groups. Also, both healthy control (HC) groups showed left frontal cortex (in middle frontal gyrus, inferior frontal gyrus, and precentral gyrus) activation in all runs, and left parietal cortex activation in post- intervention runs. The SP groups had lesions in these regions, therefore, they were masked-out in their analysis. Regarding deactivation, it was mainly found in the precuneus and posterior cingulate cortex, as observed in HC groups before intervention, and after intervention in SP who received semantic strategy organization training (SOST) and HC who received psychoeducation intervention (PI).

**Table 2:** Description of clusters found in fMRI analyses.

**Analysis/Regions within cluster**	**MNI Coordinates (x, y, z)**	**Cluster *p*-value**	**Voxels**
PI HC PRE—Activation			
Left Lateral Occipital Cortex, Left Occipital Fusiform Gyrus, Left Inferior Temporal Gyrus	−36, −86, 6	1.07E−06	1,668
Left Precentral Gyrus, Left Middle Frontal Gyrus, Left Inferior Frontal Gyrus	−52, 0, 40	1.22E−04	1,062
PI HC PRE—Deactivation			
Right Posterior Cingulate Cortex, Bilateral Precuneus, Left Cuneus, Left Occipital Pole	4, −50, 34	3.38E−03	695
SOST HC PRE—Activation			
Left Lateral Occipital Cortex, Left Occipital Fusiform Gyrus, Left Inferior Temporal Gyrus, Temporal Occipital Fusiform Gyrus, Left Occipital Pole	−34, −82, −12	3.37E−11	2,414
Left Precentral Gyrus, Left Middle Frontal Gyrus, Left Inferior Frontal Gyrus, Left Insular Cortex, Left Thalamus, Left Putamen	−46, −6, 48	4.97E−10	2,085
Right Lateral Occipital Cortex, Right Temporal Occipital Gyrus, Right Occipital Fusiform Gyrus, Right Occipital Pole, Right Cerebellum	30, −88, 2	1.22E−08	1,715
Bilateral Superior Frontal Gyrus, Bilateral Anterior Cingulate Cortex	0, −6, 74	7.17E−05	844
Cerebellar Vermis	8, −78, −30	4.15E−04	694
SOST HC PRE—Deactivation			
Bilateral Precuneus (more evident in the Right hemisphere), Right Posterior Cingulate Cortex	8, −56, 24	1.53E−03	589
PI SP PRE—Activation			
Left Lateral Occipital Cortex, Left Temporal Occipital Fusiform Gyrus, Left Occipital Fusiform Gyrus, Left Inferior Temporal Gyrus, Left Occipital Pole	−40, −78, 0	3.39E−05	1,263
SOST SP PRE—Activation			
Left Lateral Occipital Cortex, Left Occipital Fusiform Gyrus, Left Occipital Pole	−18, −100, −12	6.57E−05	953
SOST SP PRE—Deactivation			
Right Lingual Gyrus, Right Temporal Occipital Fusiform Gyrus, Right Occipital Fusiform Gyrus	26, −68, −4	4.22E−02	379
PI HC POST—Activation			
Left Precentral Gyrus, Left Middle Frontal Gyrus, Left Inferior Frontal Gyrus, Left Frontal Pole	−54, 2, 48	3.39E−09	1,906
Left Lateral Occipital Cortex, Left Occipital Fusiform Gyrus, Left Inferior Temporal Gyrus, Temporal Occipital Fusiform Gyrus	−30, −88, 0	1.35E−08	1,746
Left Supramarginal Gyrus, Borders of left intraparietal sulcus, Left inferior Parietal Cortex, Left Superior Parietal Cortex	−32, −56, 40	4.17E−06	1,133
Right Lateral Occipital Cortex, Right Occipital Pole	16, −98, 0	1.10E−03	629
PI HC POST—Deactivation			
Bilateral Precuneus, Right Posterior Cingulate Cortex	18, −56, 22	1.73E−03	593
Right Angular Gyrus, Right Inferior Parietal Cortex	54, −52, 22	3.65E−02	366
SOST HC POST—Activation			
Left Lateral Occipital Cortex, Left Occipital Fusiform Gyrus, Left Inferior Temporal Gyrus, Temporal Occipital Fusiform Gyrus, Left Occipital Pole	−30, −94, −2	8.34E−07	1,808
Borders of Left Intraparietal Sulcus, Left Superior Parietal Cortex, Left Inferior Parietal Cortex, Left Supramarginal Gyrus, Left Angular Gyrus	−26, −64, 42	1.13E−03	859
Left Precentral Gyrus, Left Middle Frontal Gyrus, Left Inferior Frontal Gyrus	−44, 4, 44	3.23E−03	741
Right Lateral Occipital Cortex, Right Occipital Fusiform Gyrus, Right Occipital Pole	28, −96, 6	4.21E−02	477
PI SP POST—Activation			
Left Lateral Occipital Cortex, Left Temporal Occipital Fusiform Gyrus, Left Occipital Fusiform Gyrus, Left Temporal Fusiform Gyrus, Left Inferior Temporal Gyrus, Left Occipital Pole	−24, −94, −4	9.57E−11	2,370
Right Lateral Occipital Cortex, Right Temporal Occipital Fusiform Gyrus, Right Occipital Fusiform Gyrus, Right Temporal Fusiform Gyrus, Right Inferior Temporal Gyrus, Right Occipital Pole	34, −88, −2	1.61E−06	1,238
Borders of Right intraparietal sulcus, Right Superior Parietal Cortex, Right Angular Gyrus, Right Inferior Parietal Cortex	34, −52, 56	5.17E−04	692
SOST SP POST—Activation			
Right Lateral Occipital Cortex, Right Occipital Pole	30, −94, 2	1.47E−05	1,012
Left Temporal Occipital Fusiform Gyrus, Left Inferior Temporal Gyrus, Left Occipital Fusiform Gyrus, Bilateral Cerebellum	−38, −70, −24	9.55E−05	837
Left Lateral Occipital Cortex, Left Occipital Pole, Left Occipital Fusiform Gyrus	−38, −92, −10	2.99E−04	736
Bilateral Supplementary Motor Area	4, 6, 64	3.35E−04	726
Right Cerebellum	38, −56, −22	1.77E−02	412
SOST SP POST—Deactivation			
Bilateral Precuneus, Bilateral Posterior Cingulate Cortex	−6, −52, 56	3.55E−05	928
SP ANOVA—Run X Intervention Interaction effect
Bilateral Ventromedial Prefrontal Cortex	−8, 44, −8	1.73E−02	461

*Clusters found in activation and deactivation analyses of fMRI tasks for each group in each run (top). Clusters found in ANOVA with stroke patient (SP) groups. PI, Psychoeducation Intervention; SOST, Semantic Organization Strategy Training; HC, Healthy Controls; SP, Stroke Patients*.

#### ANOVAs

**Figure 6:**
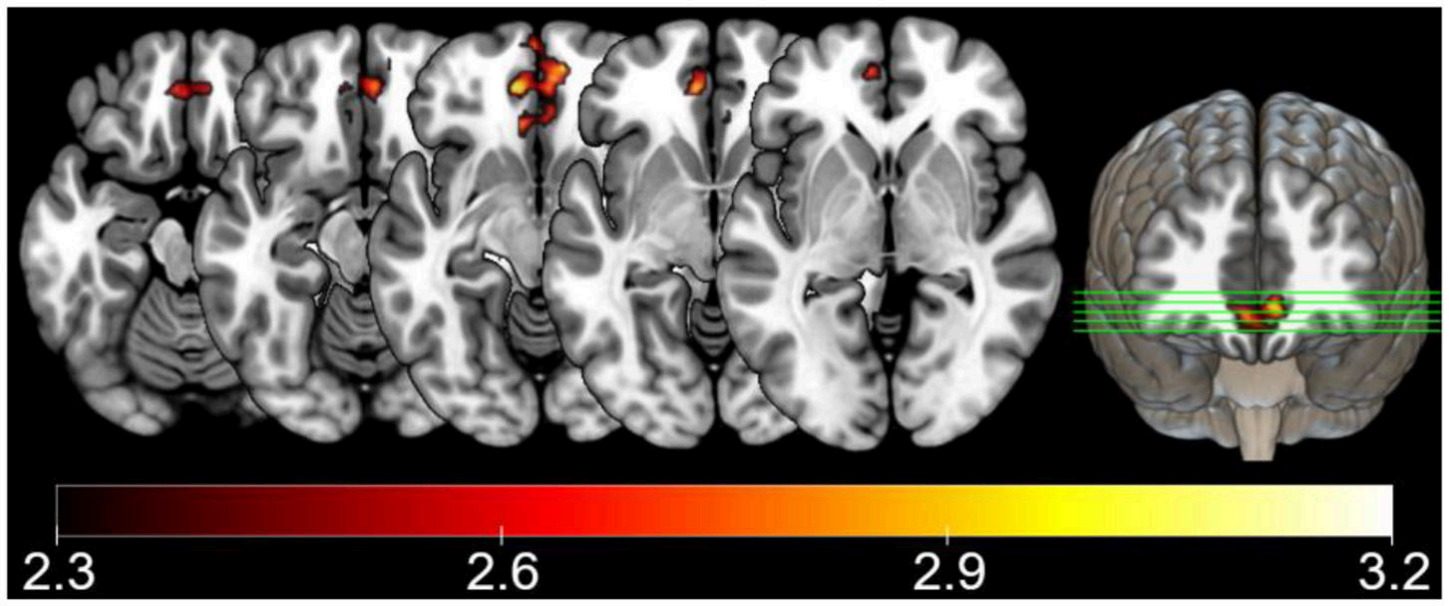
Stroke patient ANOVA results, showing significant interaction effects between run, and intervention presented as *Z-*values (neurological orientation). Voxel threshold of *Z* > 2.3 and cluster corrected threshold of *p* < 0.05.

**Figure 7:**
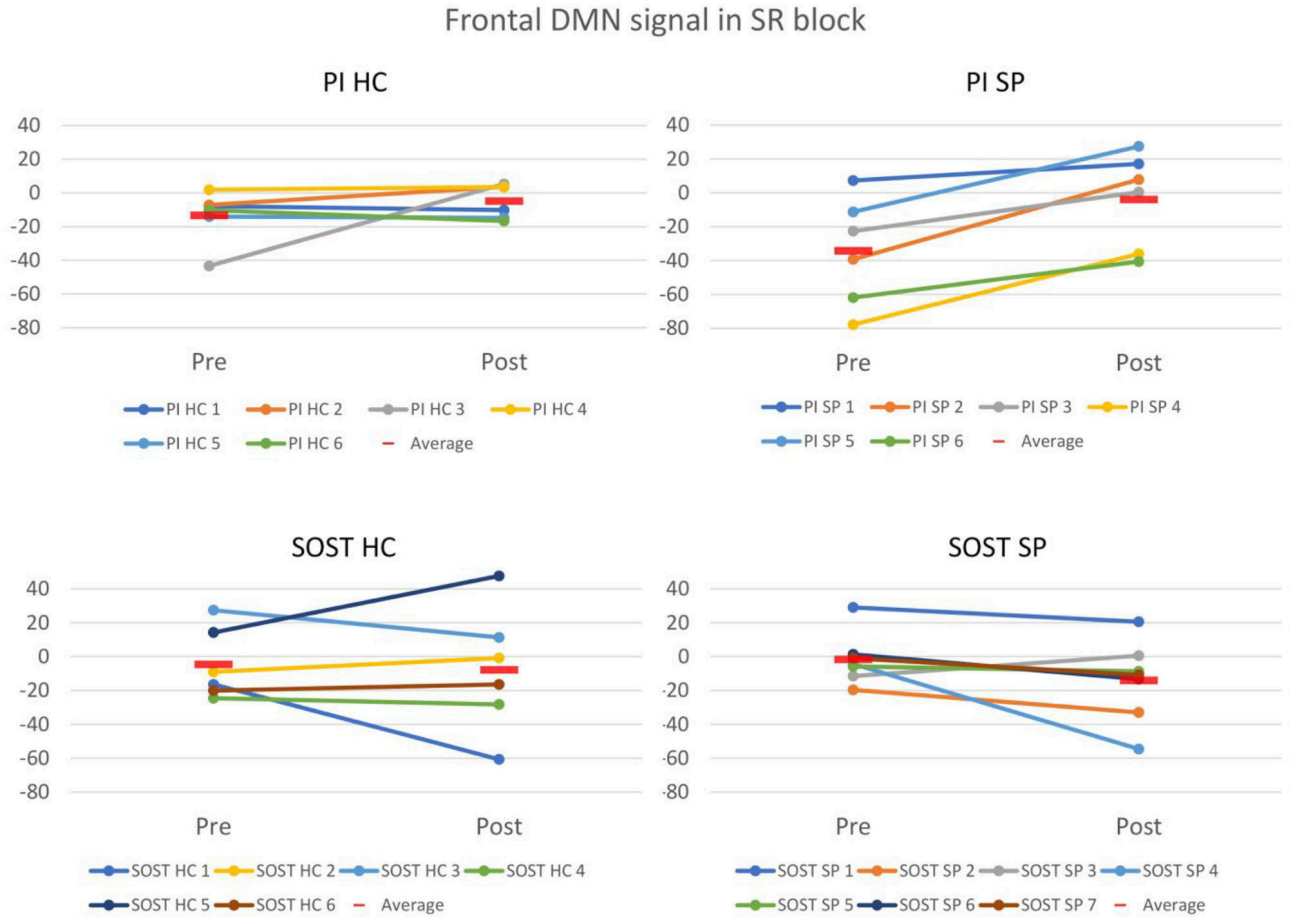
Plots of the semantically related (SR) task block BOLD response (beta-values from first-level GLM analyses) in pre and post-intervention runs in frontal cluster of default-mode network (DMN) found in the ANOVA with stroke patients (SP), showing changes for each subject, in each group. PI, Psychoeducation Intervention; SOST, Semantic Organization Strategy Training; HC, Healthy Controls; SP, Stroke Patients.

There were no significant results in the ANOVAs considering all volunteers together, which evaluated group and intervention effects, based on the outcome of a fixed-effects post-pre intervention difference analyses for each subject. However, the ANOVA performed with stroke patients (SP) evaluating run and the interaction between run and type of intervention, revealed a significant run and intervention type interaction effect in ventromedial prefrontal cortex (Table 2 and Figure 6). While the SP volunteers that received psychoeducation intervention (PI) seemed to have higher BOLD responses in SR block after intervention than before intervention, the SP who received semantic organization strategy training had the opposite effect, lower signal after intervention (Figure 7).

## Discussion

This study aimed to investigate the cognitive and behavioral outcomes and neural correlates of SOST intervention in patients with left frontoparietal ischemic stroke and healthy controls and to compare the effects of this intervention with an active control intervention (PI). The main cognitive and behavioral results demonstrated significant improvements in verbal episodic memory performance at post-intervention in patients and controls that received cognitive training (SOST) when compared to pre-intervention evaluation. These results were indicated by several scores, including the number of words recalled from the list learning paradigm (WLLP); number of consecutive words recalled from the same category, indicating a more effective application of the semantic categorization strategy; and similar scores for the SGT, the transfer and generalization task. These findings suggest that participants who received SOST improved their verbal episodic memory performance and this improvement was associated with increased semantic categorization strategy application. Similar episodic memory improvements were found in previous studies using this cognitive semantic categorization strategy in different population samples, including patients who underwent left frontal glioma removal, mild cognitive impairment, and healthy adults (13–16). Nevertheless, none of these studies included a generalization task to investigate possible near transfer effects of the cognitive training. In the current study, the group of patients and controls that received SOST intervention showed significant transfer effects demonstrated by improved SGT and semantic categorization strategy scores.

Further, the increase in strategy use after SOST intervention indicates that the improvement in task performance could be associated with the application of strategies. Also, even though there was no significant effect for the ANOVA with capacity scale (derived from the complaints questionnaire), the capacity score improvement was correlated with an increase in strategy use. This indicates that participants who used the trained strategy indeed demonstrated less memory complaints, showing a general improvement. Alternatively, since the strategy and capacity scales were not restricted to semantic organization strategy, participants that benefited from cognitive training might have been more prone to or capable of applying strategies in general, or at least to report this. Either way, it is important to notice that after receiving SOST, participants improved their performance in the generalization task and use of strategies, indicating its potential relevance particularly for stroke patients with episodic memory complaints secondary to executive function deficits related to organizational and semantic strategy application.

Although both patients and controls who received SOST showed memory improvements, the SP group could have been more directly benefited since they present more cognitive complaints than healthy controls. The fMRI results showed that the interventions had different effects only on stroke patients for the SR encoding task block. While SP who received SOST had a tendency to higher deactivation of ventromedial prefrontal cortex, SP who received PI had the opposite tendency. This region is part of the default-mode network (DMN). Indeed, the contrast used in the fMRI data analysis compared the SR blocks with the baseline condition. This comparison offers a wider vision of the impact of SOST in cerebral plasticity than comparing SR with NW block, as it is not specific to the memory and semantic differences between conditions, but includes attention and visual processing as well.

The DMN is a network negatively correlated with task-positive networks (20). Also, different studies have demonstrated higher deactivation of the DMN associated to successful memory performance, with or without cognitive training, suggesting that this brain network is highly implicated in suppressing “mind wandering” and self-reflective thoughts that can reduce external cognitive task efficiency (16, 30–34). It should be noted that in the current study, only stroke patients with lesions in the left frontoparietal areas were included. These areas are known to participate in executive networks with impact on the efficient ability to encode and recall new verbal information (17–21, 34). Indeed, during the WLLP task, the HC groups had activations in all runs in left frontal regions, and also in left parietal regions after interventions. This could explain why the control groups did not show intervention effects in brain activity during fMRI task. The preserved activity in left frontal regions along with less memory complaints usually observed in healthy controls suggest that memory improvement in the control groups would not require a specific compensatory activity, as would be the case for patients. Therefore, proper deactivation of the DMN might be important for patients with lesions in left frontoparietal areas, in order to efficiently perform this type of tasks, possibly as a compensation mechanism. As mentioned above, this might not be specific to memory task, but also related to other cognitive abilities that are important for memory. Further studies are required to evaluate the specificity of the DMN alterations to the training and to memory improvements.

The current study has some limitations, pointing to specific directions to be addressed in future studies. The specificity of the stroke lesions and the thorough screening limited the availability of volunteers. In the current study, the localization of the lesion was prioritized over the sample size in order to explore more specific processes related to left frontoparietal brain network ischemic injuries. The small sample is a possible reason for the non-significant results in fMRI ANOVA with both HC and SP, despite the significant results for ANOVA only with SP groups. This indicates that more studies with larger samples addressing this topic are required to confirm or expand our results and interpretation of the current results should not generalize to all left hemisphere stroke patients. Given this small sample, a voxel threshold of *Z* > 2.3 and a cluster corrected threshold of *p* < 0.05 were adopted. Considering sample size, statistical power and false-negative control, we believe that the threshold adopted provides balance between false-positives and false-negatives. In addition, it could be argued that there might have been retesting effects, despite the use of different stimuli in all behavior and fMRI tasks pre and post-intervention. However, retesting effects would not explain the intervention effects found in the analyses, given that they indicated higher improvement in SOST than in PI groups, and retesting effects would probably be equal in both groups. One last limitation intrinsic to studies involving patients with brain lesions is that we evaluated common effects only. Although their lesions were circumscribed to limited areas in the left hemisphere, they varied to a certain degree in extension and it is possible that in these cases different compensation mechanisms occurred. However, there were no lesion size and volume differences between the SOST and PI groups of patients, indicating that indeed the intervention type was the main difference between these groups.

## Conclusions

In conclusion, the results of the present study suggest that the SOST intervention had significant effects on cognitive and behavior performance for participants in general and a possible functional compensation for patients with stroke lesions. This was observed in areas associated with the DMN which are important for successful cognitive performance.

## Data Availability Statement

The raw data supporting the conclusions of this article will be made available by the authors, without undue reservation.

## Ethics Statement

The studies involving human participants were reviewed and approved by this study was approved by the local ethics committee (CAPPesq 8839) and ClinicalTrials.gov Identifier: NCT03644290. The patients/participants provided their written informed consent to participate in this study.

## Author Contributions

EM and MM contributed to experimental design. MM, EM, and PB contributed to data analysis and data interpretation. AB and IA contributed to data acquisition. All the authors were involved in the writing of the manuscript.

## Funding

This study was financed in part by the Coordenação de Aperfeiçoamento de Pessoal de Nível Superior – Brasil (CAPES/PROEX) - Finance Code N.: 23038.018285/2019-21/PROEX PPGN/FMUSP.

## Conflict of Interest

The authors declare that the research was conducted in the absence of any commercial or financial relationships that could be construed as a potential conflict of interest.
