# Corrigendum: Behavioral and Neural Correlates of Cognitive Training and Transfer Effects in Stroke Patients

**DOI:** 10.3389/fneur.2021.785008

**Published:** 2021-11-03

**Authors:** Eliane C. Miotto, Paulo R. Bazán, Alana X. Batista, Adriana B. Conforto, Eberval G. Figueiredo, Maria da Graça M. Martin, Isabella B. Avolio, Edson Amaro, Manoel J. Teixeira

**Affiliations:** ^1^Department of Neurology, University of São Paulo, São Paulo, Brazil; ^2^Institute of Radiology, LIM-44, University of São Paulo, São Paulo, Brazil

**Keywords:** stroke, cognitive training, episodic memory, semantic organization strategies, fMRI

In the original article, we neglected to include the funding information. The corrected Funding statement is shown below.

This study was financed in part by the Coordenação de Aperfeiçoamento de Pessoal de Nível Superior – Brasil (CAPES/PROEX) - Finance Code N.: 23038.018285/2019-21/PROEX PPGN/FMUSP.

The authors apologize for this error and state that this does not change the scientific conclusions of the article in any way. The original article has been updated.

## Publisher's Note

All claims expressed in this article are solely those of the authors and do not necessarily represent those of their affiliated organizations, or those of the publisher, the editors and the reviewers. Any product that may be evaluated in this article, or claim that may be made by its manufacturer, is not guaranteed or endorsed by the publisher.

